# Patient-Reported Status and Heart Failure Outcomes in Asia by Sex, Ethnicity, and Socioeconomic Status

**DOI:** 10.1016/j.jacasi.2023.03.015

**Published:** 2023-06-06

**Authors:** Claire A. Lawson, Wan Ting Tay, Mark Richards, Francesco Zaccardi, Jasper Tromp, Tiew-Hwa Katherine Teng, Chung-Lieh Hung, Chanchal Chandramouli, Gurpreet S. Wander, Wouter Ouwerkerk, Kanako Teramoto, Mohammad Ali, Umesh Kadam, Simon Hand, Mary Harrison, Inder Anand, Ajay Naik, Iain Squire, Kamlesh Khunti, Anna Stromberg, Carolyn S.P. Lam, ASIAN-HF Investigators

**Affiliations:** a Department of Cardiovascular Sciences, University of Leicester, Leicester, United Kingdom; b National Institute for Health Research (NIHR) Biomedical Research Centre, University of Leicester, Leicester, United Kingdom; c Leicester Real World Evidence Unit, Leicester, United Kingdom; d NIHR Applied Research Collaboration–East Midlands, University of Leicester, Leicester, United Kingdom; e National Heart Centre Singapore, Singapore; f Yong Loo Lin School of Medicine, National University of Singapore, Singapore; g Christchurch Heart Institute, University of Otago, Dunedin, New Zealand; h National University Heart Centre, Singapore; i Diabetes Research Centre, Leicester, United Kingdom; j Duke-National University of Singapore Medical School, Singapore, Singapore; k Saw Swee Hock School of Public Health, National University of Singapore and National University Health System, Singapore; l School of Allied Health, University of Western Australia, Australia; m Department of Cardiology, MacKay Memorial Hospital, Taipei, Taiwan; n Department of Cardiology, Hero Dayanand Medical College Heart Institute, Dayanand Medical College and Hospital, Ludhiana, Punjab, India; o Department of Dermatology, Amsterdam University Medical Center, University of Amsterdam, Amsterdam Infection and Immunity Institute, Amsterdam, the Netherlands; p Department of Health Sciences, University of Leicester, Leicestershire, United Kingdom; q Cardiovascular Medicine, University of Minnesota, Minneapolis, Minnesota, USA; r Care Institute of Medical Sciences, Ahmedabad, India; s Department of Health, Medicine and Caring Sciences, and Department of Cardiology, Linkoping University, Sweden; t George Institute for Global Health, Sydney, Australia; u Department of Cardiology, University of Groningen, Groningen, the Netherlands

**Keywords:** health, heart failure, hospitalization, mortality, signs, symptoms

## Abstract

**Background:**

In heart failure (HF), symptoms and health-related quality of life (HRQoL) are known to vary among different HF subgroups, but evidence on the association between changing HRQoL and outcomes has not been evaluated.

**Objectives:**

The authors sought to investigate the relationship between changing symptoms, signs, and HRQoL and outcomes by sex, ethnicity, and socioeconomic status (SES).

**Methods:**

Using the ASIAN-HF (Asian Sudden Cardiac Death in Heart Failure) Registry, we investigated associations between the 6-month change in a “global” symptoms and signs score (GSSS), Kansas City Cardiomyopathy Questionnaire overall score (KCCQ-OS), and visual analogue scale (VAS) and 1-year mortality or HF hospitalization.

**Results:**

In 6,549 patients (mean age: 62 ± 13 years], 29% female, 27% HF with preserved ejection fraction), women and those in low SES groups had higher symptom burden but lower signs and similar KCCQ-OS to their respective counterparts. Malay patients had the highest GSSS (3.9) and lowest KCCQ-OS (58.5), and Thai/Filipino/others (2.6) and Chinese patients (2.7) had the lowest GSSS scores and the highest KCCQ-OS (73.1 and 74.6, respectively). Compared to no change, worsening of GSSS (>1-point increase), KCCQ-OS (≥10-point decrease) and VAS (>1-point decrease) were associated with higher risk of HF admission/death (adjusted HR: 2.95 [95% CI: 2.14-4.06], 1.93 [95% CI: 1.26-2.94], and 2.30 [95% CI: 1.51-3.52], respectively). Conversely, the same degrees of improvement in GSSS, KCCQ-OS, and VAS were associated with reduced rates (HR: 0.35 [95% CI: 0.25-0.49], 0.25 [95% CI: 0.16-0.40], and 0.64 [95% CI: 0.40-1.00], respectively). Results were consistent across all sex, ethnicity, and SES groups (interaction *P* > 0.05).

**Conclusions:**

Serial measures of patient-reported symptoms and HRQoL are significant and consistent predictors of outcomes among different groups with HF and provide the potential for a patient-centered and pragmatic approach to risk stratification.

Heart failure (HF) prevalence is projected to increase by 40% by 2035,^1^ with the fastest rates of increase in developing countries.^2^ The prevalence of HF in some parts of Asia is 2 to 3 times that found in Europe and United States, with patients presenting as much as 20 years younger.^3^ HF is associated with multiple comorbidities, high hospitalization and mortality rates,^4^^,^^5^ and profoundly worse health-related quality of life (HRQoL) when compared to the healthy population and to many other chronic diseases.^6^, ^7^, ^8^

Current guidelines recommend regular and systematic monitoring of clinical status to optimize treatments, facilitate early recognition of clinical change, and improve outcomes.^9^ Several techniques routinely used in clinical practice to monitor HF clinical status include assessing the patients’ signs and symptoms, functional status, and exercise capacity as well as measurement of biomarkers. Prior evidence shows that changes in self-reported functional status and HRQoL closely align with symptom changes^10^ and more closely parallel physician assessments of change in clinical status than the NYHA classification, exercise capacity, weight change, or B-type natriuretic peptide level,^11^ and there have been recent calls to integrate serial recordings of HRQoL into interventional trials^12^ and routine clinical care.^13^

A key challenge to using patient-reported measures in prognosis is that patients’ experiences of symptoms and health may differ according to social or cultural characteristics. Evidence on patient-reported health status in different groups with HF is limited, but recent reports have shown that HRQoL differs by socioeconomic status (SES),^14^ country,^15^ and ethnicity.^16^, ^17^, ^18^ Although evidence over 2 decades has shown a clear association between baseline symptoms, signs,^19^^,^^20^ HRQoL,^21^, ^22^, ^23^, ^24^, ^25^, ^26^, ^27^, ^28^ and outcomes in HF, there is emerging evidence that its relationship with outcomes may also be moderated by country of origin^15^ and ethnicity.^29^ However, evidence on changing clinical status in different HF subgroups has not been evaluated.

Using a multinational, multiethnic HF registry, the objective of this study was to examine the association between different measures of clinical status change with 1-year hospitalization or death and to determine the influence of sex, ethnicity, and socioeconomic status on these associations using serial measures of: 1) signs and symptoms; 2) multidimensional HF-specific HRQoL; and 3) single-item global HRQoL in the prospective ASIAN-HF (Asian Sudden Cardiac Death in Heart Failure) Registry. Despites differences in baseline HRQoL among groups, change in HRQoL is standardized to the patient, so we hypothesized that change in HRQoL would be a consistent predictor of outcomes among different sociodemographic and ethnic groups with HF.

## Methods

### Population and setting

ASIAN-HF is a multinational prospective observational registry of Asian patients over the age of 18 years. All patients had symptomatic HF and at least 1 episode of HF decompensation in the prior 6 months requiring hospitalization or treatment with intravenous diuretics at an outpatient clinic. This report included patients recruited from 42 medical centers covering a broad spectrum of medical, cardiology, and HF specialty units in 10 regions (Taiwan, Hong Kong, India, Malaysia, Thailand, Singapore, Indonesia, the Philippines, Japan, and Korea). Patients with HF with reduced ejection fraction (ejection fraction of <40%) were enrolled consecutively between October 1, 2012, and December 31, 2015, and patients with HF with preserved ejection fraction (ejection fraction of ≥50%) between September 9, 2013, and October 31, 2016, using uniform protocols and standardized procedures. Patients with severe valvular heart disease as the primary cause of HF or life-threatening comorbidity with a life expectancy of <1 year were excluded from the registry. Details about the ASIAN-HF registry have been published.^30^ We excluded patients who had more than 4 missing signs/symptoms at baseline. Data were collected in the registry at baseline and at the 6- and 12-monthfollow-ups and included sociodemographic variables, cardiovascular history and investigations, electrocardiography and echocardiography data, comorbidities, clinical risk factors, clinical symptoms, functional status, and HRQoL scores. Patients were censored at the first event among: 1) recurrent acute heart failure; 2) death; or 3) elapse of 1 year of follow-up.

### Data access

The data used in this study are not available to other researchers because of legal restrictions imposed by multinational jurisdictions.

### Clinical status measures

#### Symptoms and signs

HF entails multiple signs and symptoms of congestion (swelling of the ankles or abdomen, dyspnea) and fatigue.^9^ We extracted information regarding the presence or absence of 13 symptoms and signs recorded at entry to the registry and at 6 months: shortness of breath on exertion, shortness of breath at rest, reduction in exercise tolerance, nocturnal cough, orthopnea, paroxysmal nocturnal dyspnea, chest pain, elevated jugular venous pressure (JVP), S_3_ gallop, peripheral edema, pulmonary rales, hepatomegaly, and hepatojugular reflux positive. Any missing or unknown symptoms or signs were coded as absent. We created a “global” symptoms and signs score (GSSS) to provide a summary of the overall “severity” of signs and symptoms, as assessed by the physician, by summing the number of signs and symptoms for each participant. The total baseline score was then categorized by the median value (2.0) to create 2 categories (GSSS low: ≤2; GSSS high: >2). To account for the 1,193 (18%) of individuals with at least 1 missing or unknown symptom or sign, we conducted a sensitivity analysis, removing these individuals. For the change in GSSS over 6 months, we summed the symptoms and signs recorded at the 6-monthfollow-up and created 3 change categories; “no change” as a 1-point or less change, “worsening” as a >1-point increase in score, and “improvement” as a >1-point decrease in score.

#### Health-related quality of life

HRQoL is a multidimensional concept covering various physical, emotional, and social aspects. We used the Kansas City Cardiomyopathy Questionnaire overall score (KCCQ-OS) recorded at baseline and at 6 months. The KCCQ is a 23-item, self-administered questionnaire covering multiple domains in relation to health: physical function, symptoms, social function, self-efficacy, and knowledge. The overall summary score can be derived from each domain, with scores ranging from 0 (worse health possible) to 100 (best health possible). Non–English-speaking participants used certified versions of the KCCQ translated into their native languages. Patients were stratified into 4 baseline health categories from “best” to “worst” using prior literature: “best” for 76 to 100, “good” for 51 to 75, “bad” for 26 to 50, and “worst” for 0 to 25. Change in KCCQ-OS was estimated using the difference between the baseline and the 6-month measure. Clinically meaningful change in KCCQ-OS is considered to be a change of 5 points or more^11^; therefore, change over 6 months was categorized as “considerable worsening of ≥10-point change,” “mild worsening of 5 to 9 points,” “no significant change” as a <5-point change, “mild improvement of 5 to 9 points,” and “considerable improvement of ≥10-point change.”

#### Visual analogue scale

HRQoL can be assessed using multi-item validated patient-reported health questionnaires or using a single-item “global” question about health. Single-item scales correlate highly with outcomes and are easy to apply. We used a single-item visual analogue scale (VAS) to assess the current health perception at baseline and at 6 months. The VAS was administered by having the patients mark the point on a 10-cm (100-mm) line that best corresponds to their current perceived health status, with 0 reflecting “worst possible health” and 10 reflecting “perfect health.” We categorized the baseline VAS score by the median value (6.0) to create 2 categories (VAS low: <6; VAS high: ≥6). For change in VAS over 6 months, we created 3 change categories: “no change” as a 1-point change or less, “worsening” as a >1-point decrease in score, and “improvement” as a >1-point increase in score.

### Ethnicity

Participants were categorized according to their self-reported ethnicity status on entry to the register as Chinese, Indian, Malay, Japanese/Korean, or Thai/Filipino/others.

### Socioeconomic status

Low SES was defined by no formal or less than primary education and monthly household income of USD≤$1,000. High was defined as secondary education and higher or monthly household income of USD≥$1,000.

### Covariates

We considered a range of clinically important variables, including the sociodemographic factors: age, sex, geographic region (Northeast, South, and Southeast Asia), education level (none or primary, secondary, preuniversity, degree or higher), regional income level (low-, middle-, and high-income region) and monthly household income (USD<$1,000; USD$1,000-USD$2,999; USD≥$3,000); HF factors: inpatient or outpatient enrolment, NYHA functional class, heart rate, and blood pressure; medications: angiotensin-converting enzyme inhibitors, angiotensin II receptor blockers, beta blockers, mineralocorticoid receptor antagonists, diuretics, and statins; lifestyle factors: body mass index, smoking, and alcohol intake; and comorbidities: coronary artery disease, atrial fibrillation, hypertension, stroke, peripheral arterial vascular disease, chronic respiratory disease, and anemia.

### Outcomes

The primary outcome of interest was a composite of all-cause mortality or hospitalization for HF at 1 year. Our secondary outcome was a composite of all-cause mortality or any hospitalization at 1 year. Clinical outcomes were censored at 1 year or last known vital status, whichever was earlier. Those who were completely lost to follow-up were excluded from the analysis.

### Statistical analysis

Baseline characteristics are described by categories of baseline GSSS and KCCQ-OS and presented as number (percentage) for categorical variables and mean ± SD or median (IQR) for continuous variables. Groups were compared using analysis of variance, Wilcoxon rank sum test, or chi-square test, as appropriate.

Next, each sign and symptom, GSSS, KCCQ-OS, and VAS were entered in turn into a Cox model using time since entry to the ASIAN-HF register as the timescale. Univariable associations between baseline individual signs and symptoms, GSSS categories, KCCQ-OS, and VAS categories and outcomes were first estimated, followed by multivariable associations: all baseline covariates that were associated with each outcome in the unadjusted models (*P* < 0.05) were included in the multivariable Cox models. The Cox proportional hazards assumption was checked using the Schoenfeld residuals test. Given that NYHA functional class is a symptoms-based classification, we omitted NYHA functional class in the individual sign and symptom models because of collinearity. Nevertheless, we did adjust by NYHA functional class in the GSSS, KCCQ-OS, and VAS score models as a pseudo-marker of HF severity. HRs and 95% CIs are presented. Next, follow-up was landmarked at 6 months to estimate in patients who survived to 6 months the association between change in the 3 clinical status measures (GSSS, KCCQ-OS, and VAS) over the previous 6 months and the same outcome. Similar modeling was applied, using time since the 6-month landmark date as the timescale. The change models were further adjusted for the baseline health score (GSSS, KCCQ-OS, or VAS as appropriate). To test whether group characteristics (sex, SES, ethnicity) modified the association between health (baseline and change measures) and outcomes, a first-order interaction term between the health measure and group was entered into the multivariable models. Two-sided*P* values of <0.05 were considered statistically significant. The multivariable models containing each of the 3 health status measures (GSSS, KCCQ-OS, and VAS) were compared using the Bayesian information criterion, which estimates prediction error, with smaller values indicating better relative quality, among different statistical models. Statistical analyses were performed using Stata 15.0 (Stata Corp).

### Ethics

Ethics approvals conforming to the Declaration of Helsinki were obtained from the relevant human ethics committees at all sites.

## Results

### Baseline characteristics

There were 6,549 patients included in the study; the mean age was 62.3 ± 13.2 years; 1,911 (29%) were female; 1,733 (26.5%) had HF with preserved ejection fraction; 4,152 (63%) had high SES; 1,394 (21%) had low SES; 1,968 (30.1%) were Chinese; 2,175 (33.2%) were Indian; 988 (15.1%) were Malay; 1,014 (15.5%) were Japanese/Korean; and 404 (6.2%)were Thai/Filipino/other (Table 1; see Supplemental Table 1 for missing data).

**Table 1:** Baseline Characteristics by Global Signs and Symptoms and HRQoL Levels

	Overall	GSSS			KCCQ Overall Score^a^				*P* Value
		GSSS Low (≤2)	GSSS High (>2)	*P* Value	76-100	51-75	26-50	0-25	
Baseline characteristics									
n	6,549	3,549	3,000		2,528	1,641	991	267	
Age, y	62.3 ± 13.2	62.3 ± 13.2	62.3 ± 13.3	0.99	62.3 ± 12.8	62.1 ± 13.7	61.9 ± 13.9	61.7 ± 14.5	0.83
Women	1,911 (29.2)	1,035 (29.2)	876 (29.2)	0.97	721 (28.5)	482 (29.4)	317 (32.0)	80 (30.0)	0.25
HFpEF	1,733 (26.5)	1,035 (29.2)	698 (23.3)	<0.001	842 (33.3)	330 (20.1)	175 (17.7)	35 (13.1)	<0.001
Geographic region				<0.001					
Northeast Asia	1,961 (29.9)	1,183 (33.3)	778 (25.9)		1,042 (41.2)	525 (32.0)	250 (25.2)	61 (22.8)	
South Asia	1,885 (28.8)	1,031 (29.1)	854 (28.5)		791 (31.3)	506 (30.8)	308 (31.1)	85 (31.8)	
Southeast Asia	2,703 (41.3)	1,335 (37.6)	1,368 (45.6)		695 (27.5)	610 (37.2)	433 (43.7)	121 (45.3)	
Regional income level				<0.001					
Low	2,284 (34.9)	1,104 (31.1)	1,180 (39.3)		867 (34.3)	644 (39.2)	448 (45.2)	128 (47.9)	
Middle	842 (12.9)	585 (16.5)	257 (8.6)		399 (15.8)	243 (14.8)	139 (14.0)	43 (16.1)	
High	3,423 (52.3)	1,860 (52.4)	1,563 (52.1)		1,262 (49.9)	754 (45.9)	404 (40.8)	96 (36.0)	
Ethnicity				<0.001					
Chinese	1,968 (30.1)	1,098 (30.9)	870 (29.0)		821 (32.5)	389 (23.7)	169 (17.1)	46 (17.2)	
Indian	2,175 (33.2)	1,168 (32.9)	1,007 (33.6)		840 (33.2)	568 (34.6)	364 (36.7)	106 (39.7)	
Malay	988 (15.1)	378 (10.7)	610 (20.3)		203 (8.0)	287 (17.5)	228 (23.0)	56 (21.0)	
Japanese/Korean	1,014 (15.5)	641 (18.1)	373 (12.4)		437 (17.3)	299 (18.2)	180 (18.2)	43 (16.1)	
Thai/Filipino/others	404 (6.2)	264 (7.4)	140 (4.7)		227 (9.0)	98 (6.0)	50 (5.0)	16 (6.0)	
Inpatient enrolment	2,377 (36.3)	752 (21.2)	1,625 (54.2)	<0.001	238 (9.4)	629 (38.3)	593 (59.8)	181 (67.8)	<0.001
NYHA functional class III/IV	1,645 (28.7)	297 (9.7)	1,348 (50.2)	<0.001	279 (13.0)	489 (32.2)	486 (54.4)	176 (72.4)	<0.001
LVEF on echocardiogram	31.0 (24.0-50.0)	33.0 (25.0-53.0)	30.0 (23.0-38.8)	<0.001	34.0 (25.0-56.0)	30.0 (24.0-37.2)	29.3 (22.0-36.0)	27.5 (22.0-35.0)	<0.001
BMI, kg/m^2^	25.5 ± 5.6	25.2 ± 5.3	25.8 ± 5.9	<0.001	25.6 ± 5.9	25.2 ± 5.2	25.3 ± 5.7	25.0 ± 5.6	0.16
Heart rate, beats/min	78.7 ± 15.7	76.7 ± 14.7	81.1 ± 16.6	<0.001	76.4 ± 13.7	78.9 ± 15.7	82.6 ± 17.9	84.5 ± 18.3	<0.001
Systolic BP, mm Hg	122.2 ± 21.3	123.2 ± 21.1	121.0 ± 21.4	<0.001	123.7 ± 20.3	121.0 ± 22.0	119.7 ± 21.7	117.8 ± 20.6	<0.001
Diastolic BP, mm Hg	72.6 ± 12.7	72.8 ± 12.5	72.4 ± 13.0	0.16	73.4 ± 12.5	72.3 ± 12.8	72.9 ± 13.4	71.7 ± 13.0	0.019
Coronary artery disease	2,995 (45.8)	1,542 (43.5)	1,453 (48.5)	<0.001	1,036 (41.0)	794 (48.4)	504 (50.9)	132 (49.4)	<0.001
Atrial fibrillation/flutter	1,272 (19.4)	683 (19.3)	589 (19.6)	0.7	454 (18.0)	325 (19.8)	206 (20.8)	50 (18.7)	0.21
Hypertension	3,799 (58.0)	1,996 (56.3)	1,803 (60.1)	0.002	1,380 (54.6)	961 (58.6)	546 (55.1)	149 (55.8)	0.081
Diabetes	2,966 (45.3)	1,508 (42.5)	1,458 (48.6)	<0.001	1,001 (39.6)	726 (44.2)	507 (51.2)	129 (48.3)	<0.001
CKD: eGFR, mL/min/1.73m^2^ < 60	2,446 (47.3)	1,137 (43.8)	1,309 (50.9)	<0.001	670 (39.0)	608 (46.3)	485 (55.6)	142 (57.5)	<0.001
Prior stroke	457 (7.0)	220 (6.2)	237 (7.9)	0.007	123 (4.9)	114 (6.9)	71 (7.2)	36 (13.5)	<0.001
Peripheral arterial vascular disease	187 (2.9)	74 (2.1)	113 (3.8)	<0.001	46 (1.8)	40 (2.4)	39 (3.9)	9 (3.4)	0.003
Chronic respiratory disease	525 (8.0)	220 (6.2)	305 (10.2)	<0.001	149 (5.9)	155 (9.4)	104 (10.5)	28 (10.5)	<0.001
Anemia	2,053 (46.8)	919 (43.7)	1,134 (49.7)	<0.001	507 (39.8)	524 (45.3)	422 (51.7)	122 (56.0)	<0.001
Smoking, ever vs never	2,466 (37.7)	1,275 (36.0)	1,191 (39.7)	0.002	831 (32.9)	638 (38.9)	413 (41.7)	119 (44.6)	<0.001
Alcohol, ever vs never	1,564 (23.9)	807 (22.8)	757 (25.2)	0.019	568 (22.5)	382 (23.3)	244 (24.6)	71 (26.6)	0.31
ACE inhibitor or ARB	4,817 (75.2)	2,653 (76.9)	2,164 (73.3)	<0.001	1,992 (81.3)	1,199 (73.8)	637 (65.3)	166 (63.4)	<0.001
Beta-blocker	4,821 (75.3)	2,738 (79.4)	2,083 (70.5)	<0.001	1,959 (80.0)	1,193 (73.5)	648 (66.5)	171 (65.3)	<0.001
MRA	3,110 (48.6)	1,682 (48.8)	1,428 (48.3)	0.74	1,196 (48.8)	817 (50.3)	513 (52.6)	124 (47.3)	0.18
Diuretics	5,078 (79.3)	2,561 (74.2)	2,517 (85.2)	<0.001	1,784 (72.8)	1,330 (81.9)	815 (83.6)	217 (82.8)	<0.001
Statin	4,139 (64.5)	2,233 (64.6)	1,906 (64.4)	0.82	1,547 (63.1)	1,004 (61.8)	597 (61.2)	141 (53.8)	0.029
Monthly household income, US$				<0.001					0.15
<1,000	3,132 (55.2)	1,692 (53.8)	1,440 (56.9)		1,418 (56.3)	920 (56.1)	534 (53.9)	154 (57.7)	
1,000-2,999	1,140 (20.1)	693 (22.0)	447 (17.7)		510 (20.2)	344 (21.0)	180 (18.2)	49 (18.4)	
≥3,000	582 (10.3)	367 (11.7)	215 (8.5)		271 (10.8)	159 (9.7)	120 (12.1)	24 (9.0)	
Decline to respond	824 (14.5)	394 (12.5)	430 (17.0)		321 (12.7)	217 (13.2)	156 (15.8)	40 (15.0)	
Highest education attained				<0.001					<0.001
None or primary	1,891 (33.3)	1,006 (32.0)	885 (35.0)		898 (35.6)	544 (33.2)	306 (30.9)	84 (31.5)	
Secondary	1,822 (32.1)	1,015 (32.3)	807 (31.9)		781 (31.0)	540 (32.9)	354 (35.8)	91 (34.1)	
Preuniversity	771 (13.6)	456 (14.5)	315 (12.4)		372 (14.8)	204 (12.4)	119 (12.0)	31 (11.6)	
Degree or higher	1,037 (18.3)	554 (17.6)	483 (19.1)		436 (17.3)	309 (18.8)	179 (18.1)	54 (20.2)	
Decline to respond	157 (2.8)	115 (3.7)	42 (1.7)		33 (1.3)	43 (2.6)	32 (3.2)	7 (2.6)	
Death in 1 year	556 (9.1)	205 (6.1)	351 (12.9)	<0.001	117 (4.9)	139 (9.4)	147 (16.6)	47 (21.0)	<0.001

Values are mean ± SD, n (%), or median (IQR).

ACE = angiotensin-converting enzyme; ARB = angiotensin receptor blocker; BMI = body mass index; BP = blood pressure; CKD = chronic kidney disease; eGFR = estimated glomerular filtration rate; GSSS = global symptoms and signs score; HFpEF = heart failure with preserved ejection fraction; HRQoL = health-related quality of life; KCCQ = Kansas City Cardiomyopathy Questionnaire; LVEF = left ventricular ejection fraction; MRA = mineralocorticoid receptor antagonists.

a KCCQ-OS available in n = 5,427 participants.

### Baseline signs, symptoms, and health

**Figure 1:**
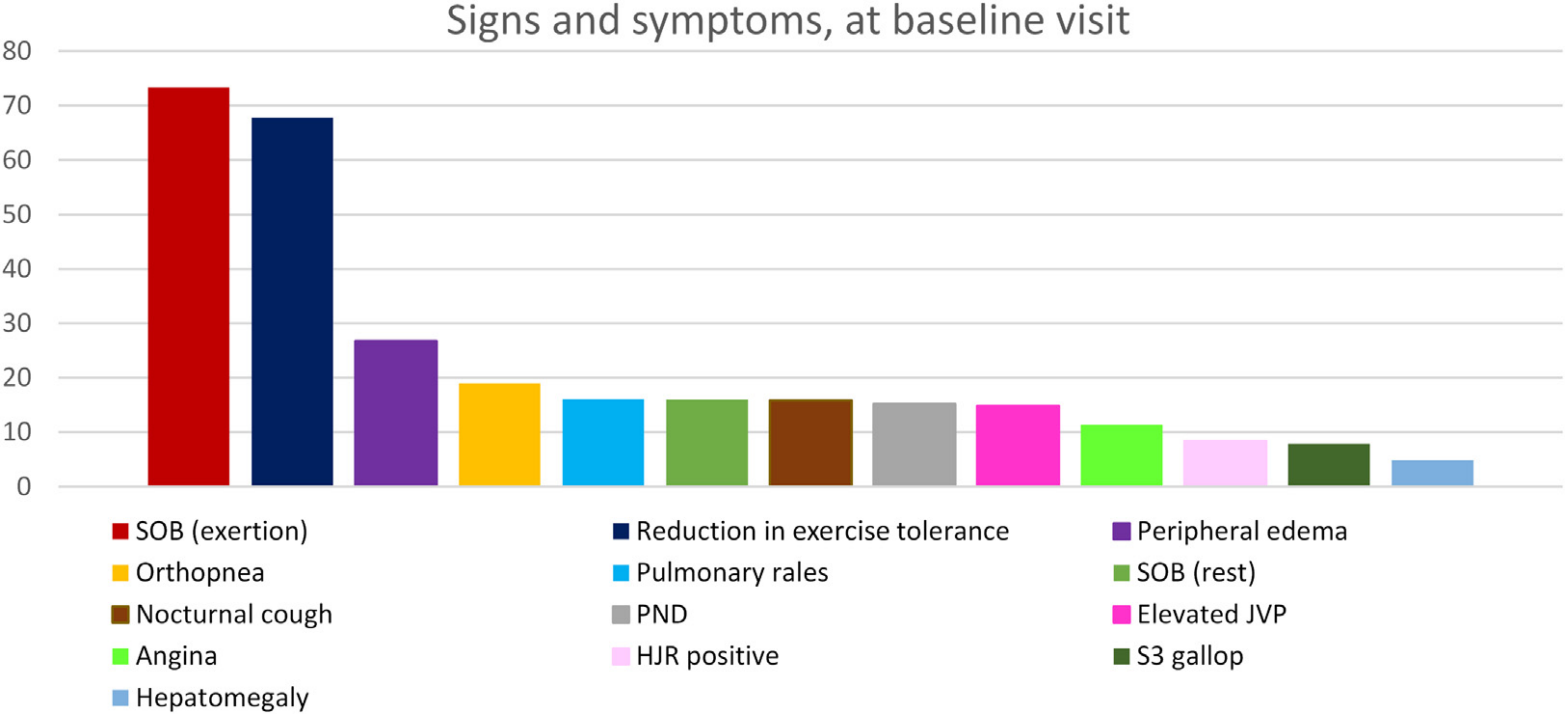
Prevalence of Signs and Symptoms at Baseline Prevalence of symptoms and signs are reported as the percentage of each symptom and sign at baseline, ranging from the most prevalent (shortness of breath) to the least prevalent (S_3_ gallop). HJR = hepatojugular reflux; JVP = jugular venous pressure; PND = paroxysmal nocturnal dyspnea; SOB = shortness of breath.

**Figure 2:**
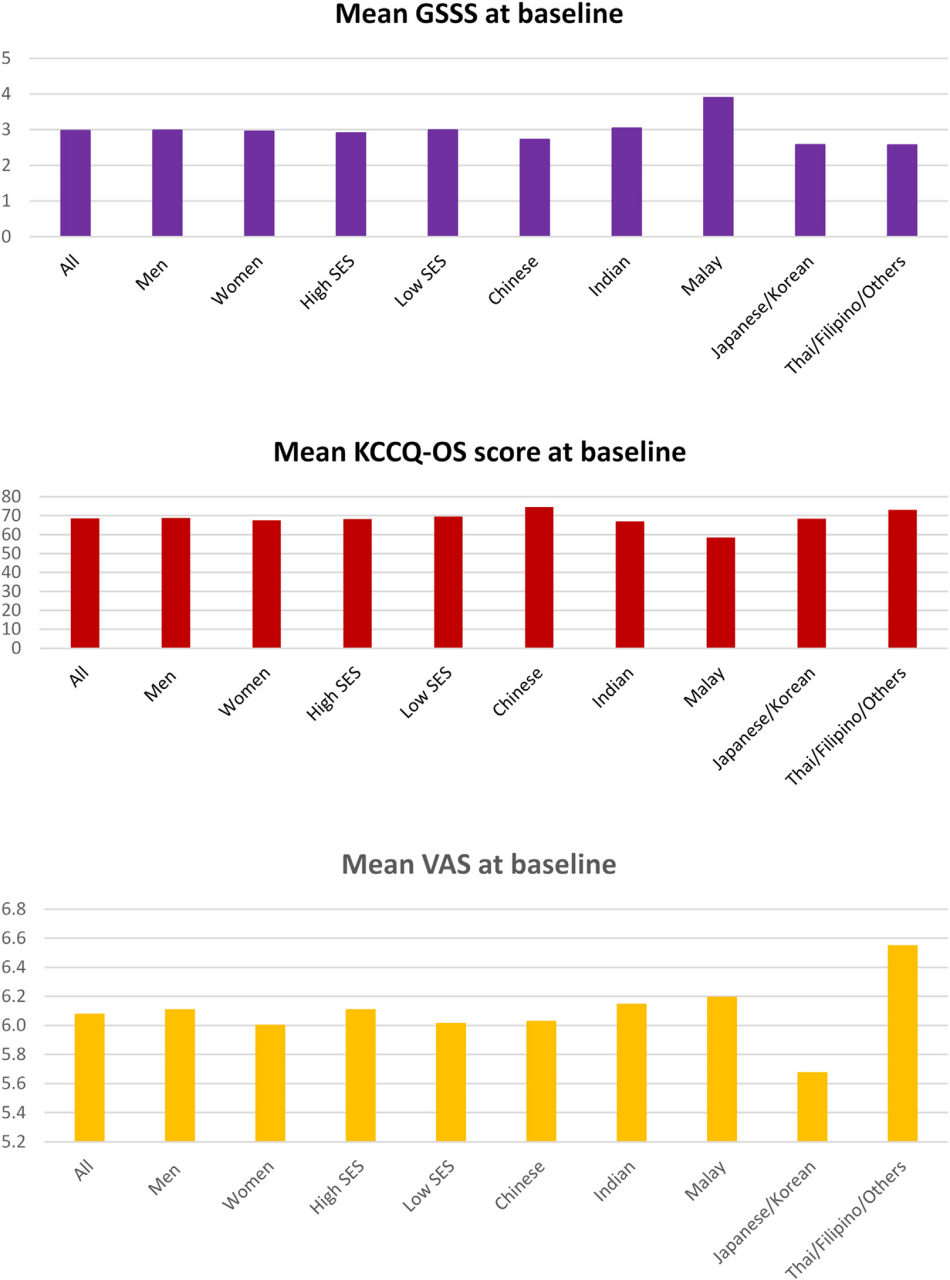
Mean GSSS, KCCQ-OS, and VAS by Sex, Socioeconomic Status, and Ethnicity Mean HRQoL scores at baseline by sex, socioeconomic status, and ethnicity. GSSS = global symptoms and signs score (range: 0-10); KCCQ-OS = Kansas City Cardiomyopathy Questionnaire overall score (range: 0 [worst] to 100 [best]); SES = socioeconomic status; VAS = visual analogue scale (range: 0 [worst] to 10 [best]).

There were 5.427 (83%) patients with KCCQ-OS and 5,521 (84%) with VAS scores at baseline. All patients had GSSS scores. The most prevalent symptoms at baseline were shortness of breath on exertion (73.4%), low exercise tolerance (67.8%), and peripheral edema (26.8%) (Figure 1). Women had a higher prevalence of all 3 symptoms than men but a lower prevalence of elevated JVP, S_3_ gallop, and hepatomegaly (Table 2). There were also differences in symptoms by SES, with the low SES group having a higher prevalence of reduced exercise tolerance, nocturnal cough, and orthopnea but a lower prevalence of elevated JVP, S_3_ gallop, and hepatomegaly than the high SES group. Overall, the mean GSSS was 3.0 ± 2.5, mean KCCQ-OS was 68.5 ± 23.0, and mean VAS was 6.1 ± 1.8. There was some borderline evidence of higher KCCQ-OS and VAS in men than in women (both *P* = 0.05), which became more marked when stratified by SES: within the high SES subgroup, men reported higher KCCQ-OS (69 vs 66; *P* = 0.0003) and VAS (6.2 vs 6.0; *P* = 0.0069) than women but no difference in GSSS. On the other hand, within the low SES subgroup, men had higher GSSS than women (3.1 vs 2.8; *P* = 0.0198) but no difference in KCCQ-OS and VAS. The differences in symptoms and overall health with the greatest magnitude were by ethnicity. Except for exercise tolerance, angina, and S_3_ gallop, Malay patients had a significantly higher prevalence of all symptoms and signs than the other groups (Table 2), the highest GSSS (3.9), and the lowest KCCQ-OS score (58.5). Japanese/Korean and Thai/Filipino/other patients had the lowest GSSS scores (2.6), followed by Chinese (2.7). Chinese patients had the highest KCCQ-OS (74.6), followed by Thai/Filipino/others (73.1) (Figure 2).

**Table 2:** Baseline Signs, Symptoms, and Health-Related Quality of Life Scores by Groups

	All	Men	Women	*P* Value	High SES^a^	Low SES^a^	*P* Value	Chinese	Indian	Malay	Japanese/Korean	Thai/Filipino/Others	*P* Value
SOB, exertion	73.4	72.3	75.9	0.003	74.7	74.9	0.893	65.8	83.2	74.6	73.6	53.5	<0.001
SOB, rest	16.0	16.0	16.0	0.987	16.6	15.8	0.504	11.8	16.9	22.0	18.4	10.6	<0.001
Low exercise tolerance	67.8	66.9	69.9	0.020	68.0	71.3	0.020	61.4	74.5	67.9	63.1	74.0	<0.001
Nocturnal cough	15.8	15.6	16.3	0.470	14.7	18.2	0.002	13.2	15.7	27.4	10.3	14.6	<0.001
Orthopnea	19.0	19.5	17.9	0.140	17.4	20.2	0.023	16.4	15.4	39.7	11.8	18.6	<0.001
PND	15.2	15.7	13.8	0.051	15.0	14.4	0.565	13.0	14.6	29.3	7.1	14.6	<0.001
Angina	11.4	11.1	12.2	0.202	12.6	12.0	0.546	12.4	12.9	11.9	6.6	9.4	<0.001
Elevated JVP	14.9	16.0	12.2	<0.001	13.6	12.2	0.171	85.6	10.3	27.9	14.1	11.6	<0.001
S_3_ gallop	7.9	8.7	6.0	<0.001	9.0	5.7	<0.001	2.1	13.1	4.9	11.6	5.7	<0.001
Peripheral oedema	26.8	25.7	29.5	0.002	24.4	28.4	0.003	34.9	18.1	38.5	20.1	22.0	<0.001
Pulmonary rales	16.1	16.4	15.3	0.259	13.1	17.1	<0.001	16.1	16.3	24.6	8.5	13.1	<0.001
Hepatomegaly	4.8	5.4	3.4	<0.001	4.7	3.4	0.041	3.0	4.8	7.9	5.9	3.7	<0.001
HJR positive	8.4	9.0	6.8	0.004	6.7	5.5	0.089	7.8	8.1	13.2	6.3	5.7	<0.001
GSSS	3.0 ± 2.5	3.0 ± 2.5	3.0 ± 2.3	0.652	2.9 ± 2.4	3.0 ± 2.4	0.261	2.7 ± 2.4	3.0 ± 2.3	3.9 ± 2.9	2.6 ± 2.3	2.6 ± 2.5	<0.001
KCCQ-OL^b^	68.5 ± 23.0	68.9 ± 22.9	67.5 ± 23.0	0.054	68.3 ± 23.1	69.5 ± 22.4	0.092	74.6 ± 21.5	67.1 ± 23.0	58.5 ± 22.3	68.4 ± 22.5	73.1 ± 22.5	<0.001
KCCQ-PL^b^	71.4 ± 25.5	72.4 ± 25.2	68.9 ± 26.3	<0.001	71.6 ± 25.3	71.3 ± 25.9	0.662	78.8 ± 23.6	67.7 ± 25.0	63.4 ± 25.8	72.0 ± 25.9	76.1 ± 25.4	<0.001
VAS^c^	6.1 ± 1.8	6.1 ± 1.8	6.0 ± 1.8	0.048	6.1 ± 1.9	6.0 ± 1.7	0.098	6.0 ± 1.7	6.1 ± 1.7	6.2 ± 1.8	5.7 ± 2.3	6.5 ± 1.9	<0.001

Values are % or mean ± SD.

GSSS = global signs and symptoms score (0-13); HJR = hepatojugular reflex; JVP = jugular venous pressure; KCCQ-OS = Kansas City Cardiomyopathy Questionnaire overall score (1-100); KCCQ-PL = Kansas City Cardiomyopathy Questionnaire Physical Limitation score (1-100); PND = paroxysmal nocturnal dyspnea; SOB = shortness of breath; VAS = visual analogue scale (1-10).

a For SES, low: less than primary education and monthly household income USD≤$1,000; high: secondary education and higher or monthly household income USD≥$1,000.

b KCCQ-OS and KCCQ-PL available for 5,427 participants.

c VAS available for 5,521 participants.

### HF admission or death

#### Baseline symptoms, signs, and health

Of the patients with baseline clinical status scores, complete follow-up was available for 6,072 (93%) GSSS, 5,066 (93%) KCCQ-OS, and 4,232 (77%) VAS patients. Each symptom and sign was significantly associated with the composite of HF admission or death within 1 year (Table 3). The strongest associations were for hepatomegaly (HR: 1.82; 95% CI: 1.47-2.24), pulmonary rales (HR: 1.74; 95% CI: 1.50-2.02), and paroxysmal nocturnal dyspnea (HR: 1.73; 95% CI: 1.48-2.02). In terms of global symptoms, a GSSS of >2 was associated with a 55% increase in the rates (HR: 1.55; 95% CI: 1.32-1.82) compared to a score of 2 or less. Results were almost identical in the sensitivity analysis after removing individuals with 1 or more unknown or missing symptom or signs (HR: 1.55; 95% CI: 1.33-1.80) (Supplemental Table 2). There was an incremental increase in the rates with each reducing category of KCCQ-OS compared to the reference category (KCCQ-OS of 76-100), ranging from an HR of 1.26 (95% CI: 1.01-1.58) for KCCQ-OS of 51 to 75 to an HR of 1.90 (95% CI: 1.37-2.63) for KCCQ-OS of 0 to 25. There was no association with VAS. Associations were independent of other clinical and patient characteristics and HF severity (indicated by NYHA functional class). They were also consistent across population groups, with no significant sex, SES, or ethnicity interactions.

**Table 3:** Outcomes by Baseline HRQoL and Change

		First Hospitalization for HF or Death			First Hospitalization for All-Cause or Death		
	N	Number of Events (%)	Unadjusted HR (95% CI)	Adjusted^a^ HR (95 CI)	Number of Events (%)	Unadjusted HR (95% CI)	Adjusted^a^ HR (95% CI)
SOB on exertion	4,429	845 (19.1)	1.35 (1.17-1.56)	1.18 (1.01-1.38)	1,116 (25.2)	1.41 (1.25-1.60)	1.29 (1.12-1.49)
SOB at rest	930	261 (28.1)	1.96 (1.70-2.25)	1.49 (1.27-1.74)	318 (34.3)	1.79 (1.58-2.03)	1.45 (1.26-1.66)
Reduced exercise tolerance	4,083	810 (19.8)	1.49 (1.30-1.71)	1.25 (1.07-1.45)	1,046 (25.7)	1.42 (1.27-1.60)	1.24 (1.09-1.41)
Nocturnal cough	930	265 (28.5)	1.98 (1.72-2.27)	1.42 (1.22-1.65)	302 (32.5)	1.65 (1.45-1.87)	1.34 (1.17-1.55)
Orthopnea	1,077	359 (33.3)	2.71 (2.39-3.08)	1.70 (1.47-1.97)	377 (35.1)	1.94 (1.72-2.18)	1.42 (1.23-1.62)
PND	853	281 (32.9)	2.52 (2.20-2.88)	1.73 (1.48-2.02)	312 (36.7)	2.02 (1.79-2.30)	1.63 (1.41-1.88)
Chest pain (angina)	655	83 (12.7)	0.67 (0.53-0.83)	0.66 (0.52-0.85)	135 (20.6)	0.86 (0.72-1.03)	0.88 (0.72-1.07)
Elevated JVP	847	299 (35.3)	2.77 (2.42-3.16)	1.48 (1.27-1.71)	304 (36.0)	1.93 (1.70-2.19)	1.28 (1.11-1.47)
S_3_ gallop	485	130 (26.8)	1.68 (1.40-2.02)	1.67 (1.36-2.04)	156 (32.4)	1.53 (1.29-1.80)	1.40 (1.17-1.68)
Peripheral edema	1572	444 (28.2)	2.24 (1.99-2.53)	1.54 (1.35-1.77)	484 (30.9)	1.64 (1.47-1.83)	1.26 (1.12-1.43)
Pulmonary rales	944	332 (35.2)	2.86 (2.51-3.25)	1.74 (1.50-2.02)	329 (35.0)	1.89 (1.67-2.14)	1.39 (1.21-1.60)
Hepatomegaly	287	108 (37.6)	2.67 (2.19-3.26)	1.82 (1.47-2.24)	115 (40.2)	2.11 (1.74-2.55)	1.44 (1.18-1.77)
Hepatojugular reflux positive	495	189 (38.2)	2.88 (2.46-3.37)	1.68 (1.41-2.00)	171 (34.6)	1.77 (1.51-2.08)	1.24 (1.04-1.47)
GSSS (n = 6,072)							
Low (≤2)	3,362	404 (12.0)	1.00 (ref)	1.00 (ref)	612 (18.2)	1.00 (ref)	1.00 (ref)
High (>2)	2,710	683 (25.2)	2.34 (2.07-2.64)	1.55 (1.32-1.82)	812 (30.0)	1.84 (1.65-2.04)	1.28 (1.11-1.47)
KCCQ-OS (n = 4,966)							
76-100	2,383	210 (8.8)	1.00 (ref)	1.00 (ref)	419 (17.6)	1.00 (ref)	1.00 (ref)
51-75	1,479	252 (17.0)	2.04 (1.70-2.45)	1.26 (1.01-1.58)	421 (28.5)	1.75 (1.53-2.00)	1.23 (1.04-1.45)
26-50	881	239 (27.1)	3.50 (2.91-4.22)	1.79 (1.40-2.28)	325 (37.1)	2.47 (2.13-2.85)	1.49 (1.24-1.80)
0-25	223	80 (35.9)	4.98 (3.85-6.44)	1.90 (1.37-2.63)	106 (47.5)	3.56 (2.88-4.40)	1.89 (1.45-2.45)
VAS (n = 5,066)							
≥6	3,177	397 (12.5)	1.00 (ref)	1.00 (ref)	648 (20.4)	1.00 (ref)	1.00 (ref)
<6	1,889	381 (20.2)	1.70 (1.48-1.96)	1.04 (0.88-1.23)	611 (32.4)	1.73 (1.55-1.93)	1.14 (1.00-1.30)
GSSS change over 6 months (n = 4,232)							
>1-point increase (worsening)	273	65 (23.8)	4.19 (3.15-5.58)	2.95 (2.14-4.06)	82 (30.0)	2.65 (2.08-3.37)	1.81 (1.38-2.37)
No significant change (–1 to 1)	2,658	171 (6.4)	1.00 (Ref)	1.00 (Ref)	345 (13.0)	1.00 (Ref)	1.00 (Ref)
>1-point decrease (improvement)	1,301	108 (8.3)	1.31 (1.03-1.67)	0.35 (0.25-0.49)	206 (15.8)	1.25 (1.05-1.49)	0.50 (0.39-0.63)
KCCQ-OS change over 6 months (n = 2,803)							
≥10-point decrease (considerable worsening)	379	51 (13.5)	1.87 (1.30-2.70)	1.93 (1.26-2.94)	69 (18.2)	1.39 (1.04-1.87)	1.47 (1.04-2.07)
5- to 9-point decrease (mild worsening)	199	15 (7.5)	1.02 (0.58-1.79)	0.82 (0.41-1.65)	25 (12.6)	0.93 (0.61-1.43)	1.00 (0.61-1.65)
No significant change (<5 points)	895	67 (7.5)	1.00 (Ref)	1.00 (Ref)	122 (13.6)	1.00 (Ref)	1.00 (Ref)
5- to 9-point increase (mild improvement)	281	15 (5.3)	0.71 (0.41-1.25)	0.52 (0.27-1.03)	30 (10.7)	0.78 (0.53-1.17)	0.72 (0.45-1.15)
≥10-point increase (considerable improvement)	1,049	52 (5.0)	0.65 (0.45-0.94)	0.25 (0.16-0.40)	117 (11.2)	0.81 (0.63-1.05)	0.48 (0.34-0.68)
VAS change over 6 months (n = 3,122)							
>1-point decrease (worsening)	351	46 (13.1)	2.28 (1.62-3.22)	2.30 (1.51-3.52)	59 (16.8)	1.57 (1.18-2.10)	1.61 (1.13-2.28)
No significant change (–1 to 1)	1,943	117 (6.0)	1.00 (Ref)	1.00 (Ref)	217 (11.2)	1.00 (Ref)	1.00 (Ref)
>1-point increase (improvement)	828	39 (4.7)	0.78 (0.55-1.13)	0.64 (0.40-1.00)	91 (11.0)	0.99 (0.78-1.26)	0.83 (0.61-1.14)

Interactions	*P* Value (First Hospitalization for HF or Death)	*P* Value (First Hospitalization for All-Cause or Death)
GSSS and sex	0.166	0.147
GSSS and SES	0.362	0.221
GSSS and ethnicity	0.661	0.161
KCCQ-OS and sex	0.991	0.388
KCCQ-OS and SES	0.084	0.627
KCCQ-OS and ethnicity	0.267	0.007
VAS and sex	0.070	0.501
VAS and SES	0.434	0.171
VAS and ethnicity	0.806	0.462
GSSS change and sex	0.225	0.979
GSSS change and SES	0.209	0.862
GSSS change and ethnicity	0.821	0.117
KCCQ-OS change and sex	0.447	0.190
KCCQ-OS change and SES	0.274	0.571
KCCQ-OS change and ethnicity	0.554	0.277
VAS change and sex	0.288	0.967
VAS change and SES	0.915	0.577
VAS change and ethnicity	0.184	0.174

The number at risk refers to the number of patients included in each analysis (those patients with follow-up data and known outcomes recorded). Ethnicity is a categorical variable including Chinese, Indian, Malay, Korean/Japanese, and Thai/Filipino/other.

Abbreviations as in Tables 1 and 2.

a Adjusted for New Yor Heart Association functional class; age; sex; ethnicity; enrollment type; regional income level; ejection fraction; systolic blood pressure; heart rate; chronic obstructive pulmonary disease; atrial fibrillation; diabetes; coronary artery disease; chronic kidney disease; peripheral arterial vascular disease; and use of angiotensin-converting enzyme inhibitors/angiotensin receptor blockers, beta blockers, or mineralocorticoid receptor antagonists (and baseline GSSS, KCCQ, or VAS for the respective change measures).

**Figure 3:**
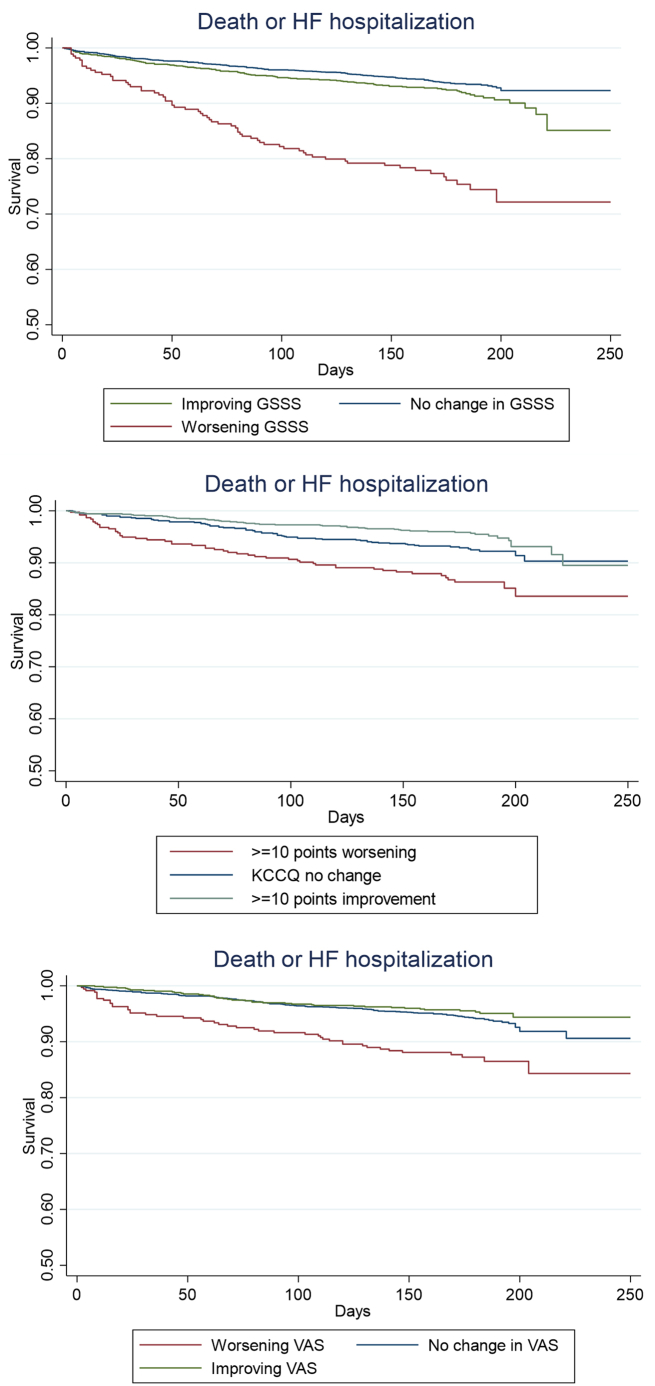
Changing Clinical Status and Admission or Mortality (Unadjusted Survival Curves) Unadjusted survival curves between change in signs, symptoms, or health-related quality of life scores and time to HF admission or death. GSSS: worsening (1-point decrease), no change (–1- to 1-point change), improving (>1-point decrease). KCCQ-OS: worsening (≥10-point decrease), no change (<5-point change), improving (≥10-point increase). VAS: worsening (>1-point decrease), no change (–1- to 1-point change), improving (>1-point increase). HF = heart failure; other abbreviations as in Figure 2.

Association of change in global signs, symptoms, and health and HF admission or death There were 306 (4.7%) patients who died and 733 (11.2%) who were lost to follow-up before the 6-month landmark point. Of the remaining 5,510 patients, baseline scores, follow-up scores, and complete follow-up were available for 4,232 (77%) GSSS, 2,803 (51%) KCCQ-OS, and 3,122 (57%) VAS patients. There were 1,301 (31%) people who experienced a reduction (improvement) in GSSS of >1 point over 6 months. Compared to the stable group with a <1-point change, this group had a 65% lower rates of HF admission or death (HR: 0.35; 95% CI: 0.25-0.49) (Table 3, Central Illustration). Conversely, compared to the same reference group, those with an increase (worsening) in GSSS of >1 point (n = 273; 6.5%) had a 3-fold increase in rates (HR: 2.95; 95% CI: 2.14-4.06). Likewise, compared to the stable reference group (<5-point change), people experiencing a ≥10-point increase (considerable improvement) in KCCQ-OS (1,049; 37%) had 75% lower rates (HR: 0.25; 95% CI: 0.16-0.40), and those experiencing a ≥10-point decrease (considerable worsening) in KCCQ-OS (379; 14%) had 93% higher rates (HR: 1.93; 95% CI: 1.26-2.94). In terms of the single-item VAS, compared to the stable group (<1-point change), people experiencing a >1-point increase (improvement) in VAS (828; 27%) had 36% lower rates (HR: 0.64; 95% CI: 0.40-1.00), and those experiencing a >1-point decrease (worsening) in VAS (n = 351; 11%) had a more than doubling of the rates (HR: 2.30; 95% CI: 1.51-3.52). These associations were independent of baseline characteristics, health status, and HF severity and consistent across population groups (see Figure 3 for unadjusted survival curves).

**Central Illustration:**
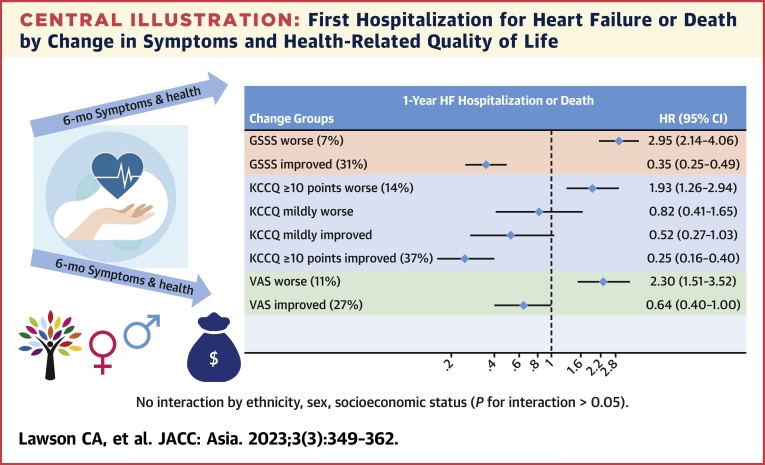
First Hospitalization for Heart Failure or Death by Change in Symptoms and Health-Related Quality of Life GSSS (1 [best] to 13 [worst]): no significant change (–1 to 1), >1-point increase (worsening), >1-point decrease (improvement). KCCQ-OS (1 [worst] to 100 [best]): no significant change (<5-point change), 5- to 9-point decrease (mild worsening), ≥10-point decrease (considerable worsening), 5- to 9-point increase (mild improvement), ≥10-point increase (considerable improvement). VAS (1 [worst] to 10 [best]): no significant change (–1 to 1), >1-point decrease (worsening), >1-point increase (improvement). SES score: low (less than primary education and monthly household income USD≤$1,000), high (secondary education and higher or monthly household income USD≥$1,000). Ethnicity: categorical variable including Chinese, Indian, Malay, Korean/Japanese, and Thai/Filipino/other. All associations adjusted for NYHA functional class, age, sex, ethnicity, enrollment type, regional income level, ejection fraction, systolic blood pressure, heart rate, chronic obstructive pulmonary disease, atrial fibrillation, diabetes, coronary artery disease, chronic kidney disease, peripheral arterial vascular disease, use of angiotensin-converting enzyme inhibitors/angiotensin II receptor blockers, beta-blockers, or mineralocorticoid receptor antagonists (and baseline GSSS, KCCQ, or VAS for the respective change measures). GSSS = global symptoms and signs score; KCCQ = Kansas City Cardiomyopathy Questionnaire; SES = socioeconomic status; VAS = visual analogue scale.

### All-cause admission or death

#### Baseline symptoms, signs, and health

Individual symptoms (eg, dyspnea, reduced exercise tolerance, cough) had significant associations with the composite of all-cause admission or death, similar to HF admission or death. Clinical signs also had significant but weaker associations (Table 3). A GSSS of >2 was associated with 28% higher rates (HR: 1.28; 95% CI: 1.11-1.47) compared to a score of 2 or less; there was an incremental increase in rates with each reducing category of KCCQ-OS ranging from an HR of 1.23 (95% CI: 1.04-1.45) for KCCQ-OS of 51 to 75 to an HR of 1.89 (95% CI: 1.45-2.43) for a KCCQ-OS of 0 to 25. Although consistent across sex and SES, there was a significant interaction between KCCQ-OS and ethnicity (interaction *P* = 0.007). Although the Thai/Filipino/other group had one of the highest mean KCCQ-OS scores at baseline, it was not associated with the composite of any admission or death in this group (Table 4). Compared to a VAS score of ≥6, a VAS score <6 was associated with 14% higher rates (HR: 1.14; 95% CI: 1.00-1.30).

**Table 4:** Association of KCCQ-OS With Composite Any-Cause Hospital Admission or Death Up to 1 Year: Interaction With Ethnicity

	Chinese (n = 810)	Indian (n = 867)	Malay (n = 471)	Japanese/Korean (n = 768)	Thai/Filipino/Others (n = 279)
KCCQ 76-100	1.00 (ref)	1.00 (ref)	1.00 (ref)	1.00 (ref)	1.00 (ref)
KCCQ 51-75	1.29 (0.98-1.71)	1.72 (1.06-2.81)	1.23 (0.79-1.92)	1.45 (1.05-2.02)	0.64 (0.35-1.19)
KCCQ 26-50	1.90 (1.35-2.67)	2.19 (1.31-3.67)	1.45 (0.89-2.36)	1.61 (1.10-2.36)	0.70 (0.28-1.71)
KCCQ 0-25	2.01 (1.21-3.34)	3.42 (1.91-6.11)	2.20 (1.12-4.30)	1.78 (0.94-3.34)	0.42 (0.08-2.13)

Values are adjusted HR (95% CI). HRs adjusted for New York Heart Association functional class; age; sex; enrollment type; regional income level; ejection fraction; systolic blood pressure; heart rate; chronic obstructive pulmonary disease; atrial fibrillation; diabetes; coronary artery disease; chronic kidney disease; PAVD; and use of angiotensin-converting enzyme inhibitors/angiotensin receptor blockers, beta blockers, or mineralocorticoid receptor antagonists.

KCCQ-OS = Kansas City Cardiomyopathy Questionnaire overall score.

#### Change in signs and symptoms and health

Compared to the stable group with <1-point change, the group experiencing a reduction of >1 point (improvement) in GSSS had 50% lower rates of all-cause admission or death (HR: 0.50; 95% CI: 0.39-0.63), and the group with a >1-point increase (worsening) in GSSS had 81% higher rates (HR: 1.81; 95% CI: 1.38-2.37). Likewise, compared to the stable reference group (<5-point change), people experiencing a ≥10-point increase (considerable improvement) in KCCQ-OS had 52% lower rates (HR: 0.48; 95% CI: 0.34-0.68), and those experiencing a ≥10-point decrease (considerable worsening) in KCCQ-OS had 47% higher rates (HR: 1.47; 95% CI: 1.04-2.07). In terms of the VAS, compared to the stable group (<1-point change), people experiencing a >1-point increase (improvement) in VAS had 27% lower rates (HR: 0.83; 95% CI: 0.61-1.14), and those experiencing a >1-point decrease (worsening) in VAS had 61% higher rates (HR: 1.61; 95% CI: 1.13-2.28). These associations were consistent across population groups.

### Comparison of measures

Baseline and serial KCCQ and VAS measures had a better model fit than the GSSS, with lower Bayesian information criterion scores (Supplemental Table 3).

## Discussion

Our findings add new information to existing evidence on the association between change in health status and outcomes in HF. In this study of ethnically and socioeconomically diverse groups of HF patients enrolled prospectively from 10 countries, we report 2 new and key findings of clinical importance. First, we found that, whereas baseline symptoms, signs, and overall self-reported HRQoL differed among population groups, they remained independent predictors of outcomes across groups. Importantly, in addition to the baseline symptoms, signs, or health status, subsequent change in clinical status was a significant predictor of poor outcomes, independent of diverse clinical characteristics and across different patient groups. Second, by using a range of serial clinical status measures, we found that the HRQoL measures, including the simple single-item VAS, had a superior model fit to the global signs and symptoms score. Our findings show that routine monitoring of patient-reported health allows for patient-centered risk stratification and may facilitate timely adjustments to management.

Physical examination and symptoms reported by patients during history taking provide a reasonable and less invasive approach to cardiovascular assessment than diagnostic tests. Our findings that symptoms and signs are associated with outcomes supports prior evidence. Although some smaller studies have shown the importance of change in congestion for outcomes in HF, the current study highlights the importance and consistency of a change in a diverse set of clinically assessed signs and symptoms among different groups of people with HF. Improvement or worsening of more than 1 sign or symptom over 6 months had independent and robust association with HF or all-cause admission or death, indicating the importance of serial clinical assessments for prognosis.

We found that overall patient-reported HRQoL and its change was a superior indicator of prognosis compared to signs and symptoms. Whereas there was a significantly higher symptom burden in women than men and in low compared to high SES groups, the overall GSSS and KCCQ-OS scores were similar across groups at baseline. This contrasts to prior reports of worse health in women and in lower SES groups compared to their respective counterparts. These differences could be explained, first, by the younger age and better HRQoL at HF presentation in the Asian population compared to African or Western European groups^15^ and, second, by the tendency of women with HF to downplay their symptoms or show higher tolerance, which may affect their self-reporting of HRQoL. There were consistent associations between HRQoL and outcomes in these groups, but there was a difference by ethnicity. In contrast to the other ethnic groups, the Thai/Filipino/other group generally had lower prevalence of symptoms and signs and better HRQoL, which was not associated with all-cause admission or death. In the SHOP (Singapore Heart Failure Outcomes and Phenotypes) study,^29^ only Chinese ethnicity had an association with mortality, and there was no association for Malay and Indian ethnic groups, which is in contrast to our findings of a significant association for all 3 groups. The number of Malay and Indian patients in the SHOP study was much lower than in our study, which may explain these differences. Our findings of the association between HRQoL within the most prevalent ethnic groups in Asia and outcomes is consistent with prior work,^18^ and we extend these findings to demonstrate the consistent and independent association between change in HRQoL and outcomes across all groups.

Most predictive models have been developed in hospital populations for mortality or for 30-day readmissions and have been shown to have poor predictive power. Risk stratification in HF often involves measuring complex, invasive clinical and biometrics data, measured at a single timepoint that fails to capture change in an individual with HF. Our findings that change in symptoms, signs, and health are consistent predictors among different patient groups adds to the prognostic information available to clinicians and potentially better identifies patients at risk of hospitalization.

Importantly, although signs and symptoms rely on clinical assessment, we found that change in patient-reported HRQoL was a significant predictor of increased risk. Furthermore, a simple 1-item HRQoL question (using the VAS) had similar model fit to a comprehensive set of questions on HF-related HRQoL for both HF and all-cause outcomes. Patient-reported outcome measures can be measured by patients and are more reproducible than clinician-assessed symptoms or even objective measures such as ejection fraction, and there is growing recognition that patient-reported outcome measures should be integrated into clinical care^13^ and have the potential for improving prognostic approaches.

### Study Strengths and limitations

To the best of our knowledge, this is the first study to investigate the importance of change in global symptoms and signs and health for prognosis in different population groups with HF. Using a multinational, multiethnic prospective observational registry–based cohort, we were able to explore the relationship between a range of repeated clinical status measures and outcomes among different population groups. We cannot rule out the potential for participation or survival bias within the ASIAN-HF registry, where healthier patients may have been more willing to participate. However, standardized protocols were used with specific language translations, training, and monitoring, and participants were representative of single country registers. By using prevalent cases of HF, we could not adjust for the duration of HF. Nevertheless, we did include severity measures in our analysis among a range of other essential adjustment factors. Some groups were small, and these findings need further investigation in larger cohorts to elucidate any differences among groups fully and interventional studies to determine the utility of HRQoL measures with or without signs and symptoms. The GSSS was developed to provide a summary of the overall “severity” of signs and symptoms as assessed by the physician, but further testing is required evaluate the individual contribution of the symptoms and signs components as well as different cutpoints. Further work is also needed to explore the influence of HF subtype. Although we adjusted for ejection fraction, we could not further stratify by subtype given the smaller numbers by group characteristics.

## Conclusions

Serial measures of self-reported symptoms and health are predictive of outcomes in HF among different groups of patients and provide the potential for a patient-centered, cost-effective, and straightforward approach to risk stratification. Health service providers should consider the routine recording of patient-reported information to guide shared decision making and care.

Perspectives **COMPETENCY IN MEDICAL KNOWLEDGE 1:** In a large study of ethnically and socioeconomically diverse groups of HF patients in Asia, worsening of patient-reported symptoms and HRQoL were associated with significantly higher risk of 1-year HF admission or death. Conversely, improvement was associated with significantly lower risk.

**COMPETENCY IN PATIENT CARE:** Serial measures of patient-reported symptoms and HRQoL are significant and consistent predictors of outcomes among different groups with HF and should be recorded in routine clinical care

**COMPETENCY IN PATIENT CARE:** Serial measures of patient-reported symptoms and HRQoL should be considered in risk stratification.

**TRANSLATIONAL OUTLOOK:** Further investigation in people with different HF subtypes is required, and the feasibility and impact of routine recording of patient-reported symptoms and HRQoL needs to be tested.

## Funding Support and Author Disclosures

The ASIAN-HF study is supported by grants from Boston Scientific Investigator Sponsored Research Program, National Medical Research Council of Singapore, A∗STAR Biomedical Research Council ATTRaCT program, and Bayer. Dr Lawson is funded by the National Institute for Health Research (NIHR) (NIHR-30011). Drs Khunti and Lawson are supported by the NIHR Applied Research Collaboration–East Midlands and the NIHR Leicester Biomedical Research Centre. Dr Tromp is supported by the National University of Singapore Start-up grant, the Tier 1 grant from the Ministry of Education, and the Clinician Scientist-Individual Research Grant New Investigator Grant from the National Medical Research Council; has received consulting or speaker fees from Daiichi-Sankyo, Boehringer Ingelheim, Roche Diagnostics, and Us2.ai; and owns patent US-10702247-B2 unrelated to the present work. Dr Khunti served as a consultant or speaker and/or received grants for investigator-initiated studies for AstraZeneca, Bayer, Novartis, Novo Nordisk, Sanofi-Aventis, Lilly, Merck Sharp & Dohme, Boehringer Ingelheim, and Bayer. Dr Lam is supported by a Clinician Scientist Award from the National Medical Research Council of Singapore; has received research support from Bayer and Roche Diagnostics; has served as consultant or on the Advisory Board/Steering Committee/Executive Committee for Abbott, Actelion, Alleviant Medical, Allysta Pharma, Amgen, AnaCardio AB, Applied Therapeutics, AstraZeneca, Bayer, Boehringer Ingelheim, Boston Scientific, Cytokinetics, Darma Inc, EchoNous Inc, Impulse Dynamics, Ionis Pharmaceutical, Janssen Research & Development LLC, Medscape/WebMD Global LLC, Merck, Novartis, Novo Nordisk, Prosciento Inc, Radcliffe Group Ltd, Roche Diagnostics, Sanofi, Siemens Healthcare Diagnostics, and Us2.ai; and serves as cofounder and nonexecutive director of Us2.ai. Dr Richards has received research support from Boston Scientific, Bayer, AstraZeneca, Medtronic, Roche Diagnostics, Abbott Laboratories, Thermo Fisher, Critical Diagnostics and has consulted for Bayer, Novartis, Merck, AstraZeneca, Roche Diagnostics. All other authors have reported that they have no relationships relevant to the contents of this paper to disclose.
