# Supplementary material for: Patient-Reported Status and Heart Failure Outcomes in Asia by Sex, Ethnicity, and Socioeconomic Status
Source: JACC Asia. 2023 Jun 6;3(3):349–62. doi: 10.1016/j.jacasi.2023.03.015 (PMC10261894; doi:10.1016/j.jacasi.2023.03.015)
Supplement: Supplemental Data [file mmc1.docx]

**Supplementary file**

**Contents**

[Tables 2](#_Toc111533606)

[Supplemental Table 1: Missing data 2](#_Toc111533607)

[Supplemental Table 2: Sensitivity analysis after removing individuals with at least one missing or unknown sign or symptom. 3](#_Toc111533608)

[Supplemental Table 3: Model fit statistics for the three clinical status scores 3](#_Toc111533609)

[Additional references (31 to 48) 4](#_Toc111533610)

[The ASIAN-HF Executive Committee 6](#_Toc111533611)

# **Tables**

## **Supplemental Table 1: Missing data**

|  |  |  |
| --- | --- | --- |
| **Baseline characteristics** | **No. observations** | **% missing** |
| N |  |  |
| Age, years | 6549 | 0.0 |
| Women | 6549 | 0.0 |
| HFpEF | 6549 | 0.0 |
| Geographical region | 6549 | 0.0 |
| Northeast Asia |  |  |
| South Asia |  |  |
| Southeast Asia |  |  |
| Regional income level | 6549 | 0.0 |
| Low |  |  |
| Middle |  |  |
| High |  |  |
| Race, recoded 5 groups | 6549 | 0.0 |
| Chinese |  |  |
| Indian |  |  |
| Malay |  |  |
| Japanese/Korean |  |  |
| Thai/Filipino/Others |  |  |
| Inpatient enrolment | 6,549 | 0.0 |
| NYHA Class III/IV | 5,737 | 12.4 |
| LVEF on Echo | 6,549 | 0.0 |
| BMI | 5,847 | 10.7 |
| Heart rate | 6,455 | 1.4 |
| Systolic BP | 6,496 | 0.8 |
| Diastolic BP | 6,496 | 0.8 |
| Coronary artery disease | 6,545 | 0.1 |
| Atrial fibrillation/flutter | 6,546 | 0.0 |
| Hypertension | 6,546 | 0.0 |
| Diabetes | 6,549 | 0.0 |
| CKD (eGFR<60) | 5,170 | 21.1 |
| Prior stroke | 6,547 | 0.0 |
| Peripheral arterial vascular disease | 6,544 | 0.1 |
| Chronic respiratory disease | 6,547 | 0.0 |
| Anemia | 4,386 | 33.0 |
| Smoking, ever vs never | 6,546 | 0.0 |
| Alcohol, ever vs never | 6,545 | 0.1 |
| ACEi or ARB | 6,404 | 2.2 |
| Beta-blocker | 6,404 | 2.2 |
| MRA | 6,404 | 2.2 |
| Diuretics | 6,404 | 2.2 |
| Statin | 6,415 | 2.0 |
| Household income, USD | 5,678 | 13.3 |
| <1000 |  |  |
| 1000-2999 |  |  |
| >=3000 |  |  |
| Decline to respond |  |  |
| Highest education attained | 5,678 | 13.3 |
| None or primary |  |  |
| Secondary |  |  |
| Pre-University |  |  |
| Degree or higher |  |  |
| Decline to respond |  |  |
| Death in 1 year | 6,086 | 7.1 |
| HFpEF, heart failure with preserved ejection fraction, NYHA, new York heart association; LVEF, left ventricular ejection fraction; BMI, body mass index; bpm, beats per minute; BP, blood pressure; CKD, chronic kidney disease; ACE, Angiotensin-converting enzyme inhibitors; ARB, Angiotensin II receptor blockers; MRA, mineralocorticoid receptor antagonists; USD, United States Dollars | | |

## **Supplemental Table 2: Sensitivity analysis after removing individuals with at least one missing or unknown sign or symptom.**

| Global signs and symptoms score (GSSS*) | | | | 1^st^ hospitalization for HF or death | |
| --- | --- | --- | --- | --- | --- |
|  | | | |  |  |
|  | **No. at risk** | | **No. of events (%)** | Unadjusted HR (95% CI) | Adjusted HR* (95% CI) |
| GSSS low (≤2) | n=2884 | 361 (12.5%) | | 1.00 (ref) | 1.00 (ref) |
| GSSS high (>2) | n=2472 | 642 (25.2%) | | 2.32 (2.04-2.64) | 1.55 (1.33-1.80) |
| * Adjusted for NYHA, age, sex, ethnicity, enrolment type, regional income level, ejection fraction, systolic blood pressure, heart rate, COPD, atrial fibrillation, diabetes, CAD, CKD, PAVD, use of ACEi/ARB, beta blockers or MRA and baseline GSSS | | | | | |
|  | | | | | |

## **Supplemental Table 3: Model fit statistics for the three clinical status scores**

|  | | 1st hospitalization  for HF or death | | 1st hospitalization  for any cause or death | | |
| --- | --- | --- | --- | --- | --- | --- |
|  | | BIC | | | BIC |  |
| **Baseline** |  | |  | | | |
| GSSS (>2 vs ≤2) | 10117.759 | | 16373.791 | | | |
| KCCQ-OS | 9682.0341 | | 15630.675 | | | |
| VAS (<6 vs ≥6) | 9656.5069 | | 15492.553 | | | |
| **Change over 6 months** |  | |  | | | |
| GSSS | 2913.177 | | 5171.196 | | | |
| KCCQ-OS | 2279.3757 | | 4163.0865 | | | |
| VAS | 2348.7954 | | 4160.6791 | | | |
| Adjusted for NYHA, age, sex, ethnicity, enrolment type, regional income level, ejection fraction, systolic blood pressure, heart rate, COPD, atrial fibrillation,  diabetes, CAD, CKD, PAVD, use of ACEi/ARB, beta blockers or MRA (and baseline GSSS, KCCQ or VAS for the respective change measures).  GSSS, global signs and symptoms score; KCCQ-OS, Kansas City Cardiomyopathy Questionnaire Overall Score; VAS, visual analogue scale, | | | | | | |

# **Additional references (31 to 48)**

31. Luo N, Teng TK, Tay WT, et al. Multinational and multiethnic variations in health-related quality of life in patients with chronic heart failure. Am Heart J. 2017 Sep;191:75-81.

32. Lam CS, Anand I, Zhang S, et al. Asian Sudden Cardiac Death in Heart Failure (ASIAN-HF) registry. Eur J Heart Fail. 2013 Aug;15(8):928-36.

33. Green C, Porter C, Bresnahan D, Spertus J. Development and evaluation of the Kansas City Cardiomyopathy Questionnaire: a new health status measure for heart failure FREE. American College of Cardiology. 2000;35(5):1245-55.

34. Joseph SM, Novak E, Arnold SV, et al. Comparable performance of the Kansas City Cardiomyopathy Questionnaire in patients with heart failure with preserved and reduced ejection fraction. Circ Heart Fail. 2013 Nov;6(6):1139-46.

35. Elder A, Japp A, Verghese A. How valuable is physical examination of the cardiovascular system? BMJ. 2016;354:i3309.

36. Drazner MH, Hellkamp AS, Leier CV, et al. Value of clinician assessment of hemodynamics in advanced heart failure: the ESCAPE trial. Circulation.Heart failure. 2008;1(3):170-7.

37. Caldentey G, Khairy P, Roy D, et al. Prognostic value of the physical examination in patients with heart failure and atrial fibrillation: insights from the AF-CHF trial (atrial fibrillation and chronic heart failure). JACC Heart Fail. 2014 Feb;2(1):15-23.

38. Lucas C, Johnson W, Hamilton MA et al. Freedom from congestion predicts good survival despite previous class IV symptoms of heart failure. Am Heart J. 2000 Dec;140(6):840-7.

39. Blumer V, Greene SJ, Wu A, et al. Sex Differences in Clinical Course and Patient-Reported Outcomes Among Patients Hospitalized for Heart Failure. JACC: Heart Failure. 2021;9(5):336-45.

40. Truby LK, O'Connor C, Fiuzat M, et al. Sex Differences in Quality of Life and Clinical Outcomes in Patients With Advanced Heart Failure: Insights From the PAL-HF Trial. Circulation.Heart failure. 2020;13(4):e006134.

41. Fonseca AF, Lahoz R, Proudfoot C, et al. Burden and Quality of Life Among Female and Male Patients with Heart Failure in Europe: A Real-World Cross-Sectional Study. Patient Prefer Adherence. 2021 Jul 30;15:1693-706.

42. Moradi M, Daneshi F, Behzadmehr R, et al. Quality of life of chronic heart failure patients: a systematic review and meta-analysis. Heart Fail Rev. 2020 Nov;25(6):993-1006.

43. Isaksson RM, Brulin C, Eliasson M, et al. Older women's prehospital experiences of their first myocardial infarction. J Cardiovasc Nurs. 2013;28(4):360-9.

44. Walsh MN, Joynt KE. Delays in Seeking Care: A Women's Problem? Circ Cardiovasc Qual Outcomes. 2016 Feb;9(2 Suppl 1):97.

45. Mahajan SM, Heidenreich P, Abbott B, et al. Predictive models for identifying risk of readmission after index hospitalization for heart failure: A systematic review. Eur J Cardiovasc Nurs. 2018 Sep 7:1474515118799059.

46. Ouwerkerk W, Voors AA, Zwinderman AH. Factors Influencing the Predictive Power of Models for Predicting Mortality and/or Heart Failure Hospitalization in Patients With Heart Failure. JACC Heart Fail. 2014 Oct;2(5):429-36.

47. Raphael C, Briscoe C, Davies J, et al. Limitations of the New York Heart Association functional classification system and self-reported walking distances in chronic heart failure. Heart. 2007 Apr;93(4):476-82.

48. Harry Hemingway, Richard D Riley, Douglas G Altman. Ten steps towards improving prognosis research. BMJ. 2009;339.

##

# **The ASIAN-HF Executive Committee**

• Professor A. Mark Richards (as Chairman), Cardiovascular Research Institute, National University of Singapore, Singapore. Email: mdcarthu@nus.edu.sg

• Professor Carolyn S.P. Lam (as Principal Investigator), National Heart Centre Singapore, Duke-NUS Medical School, Singapore. Email: carolyn.lam@duke-nus.edu.sg

• Professor Inder Anand (as Director, Publications Committee), University of Minnesota Medical School, VA Medical Center Minneapolis and San Diego, United States of America. Email: anand001@umn.edu

• Dr Chung-Lieh Hung, Mackay Memorial Hospital, Taipei, Taiwan. Email: jotaro3791@gmail.com

• Professor Lieng Hsi Ling (as Director, Echo Core Laboratory), Cardiovascular Research Institute, National University of Singapore, Singapore. Email: lieng_hsi_ling@nuhs.edu.sg

• Dr Houng Bang Liew, Queen Elizabeth II Hospital, Clinical Research Center, Sabah, Malaysia. Email: hbliew22@gmail.com

• Dr Calambur Narasimhan, Care Hospital, Hyderabad, India. Email: calambur@hotmail.com

• Dr Tachapong Ngarmukos, Ramathibodi Hospital, Mahidol University, Bangkok, Thailand. Email: tachaponis.nga@mahidol.ac.th

• Dr Sang Weon Park, SeJong General Hospital, Seoul, South Korea. Email: swparkmd@gmail.com

• Dr Eugenio Reyes, Manila Doctors Hospital, Manila, Philippines. Email: eugenereyes@yahoo.com

• Professor Bambang B. Siswanto, National Cardiovascular Center Universitas Indonesia, Jakarta, Indonesia. Email: bambbs@gmail.com

• Professor Wataru Shimizu, Department of Cardiovascular Medicine, Nippon Medical School, Tokyo, Japan. Email: wshimizu@nms.ac.jp

• Professor Shu Zhang, Fuwai Cardiovascular Hospital, Beijing, People’s Republic of China. Email: zsfuwai@vip.163.com

**COUNTRY AND SITE INVESTIGATORS**

**Hong Kong**

The Chinese University of Hong Kong: Cheuk Man Yu (Country PI).

**India**

CARE Hospital: Calambur Narasimhan (Country PI), B K S Sastry, Arun Gopi, K Raghu, C Sridevi, Daljeet Kaur. Care Institute of Medical Sciences: Ajay Naik, Keyur Parikh, Anish Chandarana, Urmil Shah, Milan Chag, Hemang Baxi, Satya Gupta, Jyoti Bhatia, Vaishali Khakhkhar, Vineet Sankhla, Tejas Patel, Vipul Kapoor. Hero Dayanand Medical College Heart Institute: Gurpreet Singh Wander, Rohit Tandon. Medanta-The Medicity: Vijay Chopra, Manoj Kumar, Hatinder Jeet Singh Sethi, Rashmi Verma, Sanjay Mittal. Sir Ganga Ram Hospital: Jitendra Sawhney, Manish Kr. Sharma. Westfort Hi-Tech Hospital Ltd: Mohanan Padinhare Purayil.

**Indonesia**

Rumah Sakit Jantung dan Pembuluh Darah Harapan Kita: Bambang Budi Siswanto (Country PI). RS Dr Hasan Sadikin: Pintoko Tedjokusumo, Erwan Martanto, Erwinanto. R S Khusus Jantung Binawaluya: Muhammad Munawar, Jimmy Agung Pambudi. RS Siloam Karawaci: Antonia Lukito, Ingrid Pardede, Alvin Thengker, Vito Damay, Siska Suridanda Danny, Rarsari Surarso.

**Japan**

Nippon Medical School: Wataru Shimizu (Country PI), National Cerebral and Cardiovascular Center: Takashi Noda, Ikutaro Nakajima, Mitsuru Wada, Kohei Ishibashi. Kinki University Hospital Cardiovascular Center: Takashi Kurita, Ryoubun Yasuoka. Nippon Medical School Hospital: Kuniya Asai, Kohji Murai, Yoshiaki Kubota, Yuki Izumi.Toho University Omori Medical Center: Takanori Ikeda, Shinji Hisatake, Takayuki Kabuki, Shunsuke Kiuchi, Tokyo Women's Medical University: Nobuhisa Hagiwara, Atsushi Suzuki, Dr. Tsuyoshi Suzuki.

Korea

SeJong General Hospital: Sang-Weon Park (Country PI), Suk Keun Hong, SookJin Lee, Lim Dal Soo, Dong-Hyeok Kim. Korea University Anam Hospital: Jaemin Shim, Seong-Mi Park, Seung-Young Roh, Young Hoon Kim, Mina Kim, Jong-Il Choi. Korea University Guro Hospital: Jin Oh Na, Seung Woon Rha, Hong Seog Seo, Dong Joo Oh, Chang Gyu Park, Eung Ju Kim, Sunki Lee,

Severance Hospital, Yonsei University Health System: Boyoung Joung, Jae-Sun Uhm, Moon Hyoung Lee, In-Jeong Cho, Hui-Nam Park. Chonnam National University Hospital: Hyung-Wook Park, Jeong-Gwan Cho, Namsik Yoon, KiHong Lee, Kye Hun Kim. Korea University Ansan Hospital: Seong Hwan Kim.

Malaysia

Hospital Queen Elizabeth II: Houng Bang Liew (Country PI), Sahrin Saharudin, Boon Cong Beh, Yu Wei Lee, Chia How Yen, Mohd Khairi Othman, Amie-Anne Augustine, Mohd Hariz Mohd Asnawi, Roberto Angelo Mojolou, You Zhuan Tan, Aida Nurbaini Arbain, Chii Koh Wong. Institut Jantung Negara: Razali Omar, Azmee Mohd Ghazi, Surinder Kaur Khelae, David S.P. Chew, Lok Bin Yap, Azlan Hussin, Zulkeflee Muhammad, Mohd. Ghazi Azmee. University Malaya Medical Centre: Imran Zainal Abidin, Ahmad Syadi Bin Mahmood Zhudi, Nor Ashikin Md Sari, Ganiga Srinivasaiah Sridhar, Ahmad Syadi Mahmood Zuhdi. Muhammad Dzafir Ismail. Sarawak General Hospital Heart Centre: Tiong Kiam Ong, Yee Ling Cham, Ning Zan Khiew, Asri Bin Said, Alan Yean Yip Fong, Nor Hanim Mohd Amin, Keong Chua Seng, Sian Kong Tan, Kuan Leong Yew.

Philippines

Manila Doctors Hospital: Eugenio Reyes (Country PI), Jones Santos, Allan Lim. Makati Medical Center: Raul Lapitan, Ryan Andal, Philippine Heart Center: Eleanor Lopez.

Singapore

National Heart Centre Singapore: Carolyn S.P. Lam (Country PI), Kheng Leng David Sim, Boon Yew Tan, Choon Pin Lim, Louis L.Y. Teo, Laura L.H. Chan. National University Heart Centre: Lieng Hsi Ling, Ping Chai, Ching Chiew Raymond Wong, Kian Keong Poh, Tan Tock Seng Hospital: Poh Shuan Daniel Yeo, Evelyn M. Lee, Seet Yong Loh, Min Er Ching, Deanna Z.L. Khoo, Min Sen Yew, Wenjie Huang. Changi General Hospital-Parent: Kui Toh Gerard Leong, Jia Hao Jason See, Yaozong Benji Lim, Svenszeat Tan, Colin Yeo, Siang Chew Chai. Singapore General Hospital-Parent: Fazlur Rehman Jaufeerally, Haresh Tulsidas, Than Aung. Khoo Teck Puat Hospital: Hean Yee Ong, Lee Fong Ling, Dinna Kar Nee Soon

Taiwan

Mackay Memorial Hospital, Taipei, Taiwan: Chung-Lieh Hung (Country PI), Hung-I Yeh,Jen-Yuan Kuo, Chih-Hsuan Yen. National Taiwan University Hospital: Juey-Jen Hwang, Kuo-Liong Chien, Ta-Chen Su, Lian-Yu Lin, Jyh-Ming Juang, Yen-Hung Lin, Fu-Tien Chiang, Jiunn-Lee Lin, Yi-Lwun Ho, Chii-Ming Lee, Po-Chih Lin, Chi-Sheng Hung, Sheng-Nan Chang, Jou-Wei Lin, Chih-Neng Hsu. Taipei Veterans General Hospital: Wen-Chung Yu, Tze-Fan Chao, Shih-Hsien Sung, Kang-Ling Wang, Hsin-Bang Leu, Yenn-Jiang Lin, Shih-Lin Chang, Po-Hsun Huang, Li-Wei Lo, Cheng-Hsueh Wu. China Medical University Hospital: Hsin-Yueh Liang, Shih-Sheng Chang, Lien-Cheng Hsiao, Yu-Chen Wang, Chiung-Ray Lu, Hung-Pin Wu, Yen-Nien Lin, Ke-Wei Chen, Ping-Han Lo, Chung-Ho Hsu, Li-Chuan Hsieh.

Thailand

Ramathibodi Hospital: Tachapong Ngarmukos (Country PI), Mann Chandavimol, Teerapat Yingchoncharoen, Prasart Laothavorn. Phramongkutklao Hospital:Waraporn Tiyanon. Maharaj Nakorn Chiang Mai Hospital: Wanwarang Wongcharoen, Arintaya Phrommintikul.
